# Regulatory T cells inhibit CD34+ cell differentiation into NK cells by blocking their proliferation

**DOI:** 10.1038/srep22097

**Published:** 2016-02-26

**Authors:** Isabela Pedroza-Pacheco, Divya Shah, Anna Domogala, Martha Luevano, Michael Blundell, Nicola Jackson, Adrian Thrasher, Alejandro Madrigal, Aurore Saudemont

**Affiliations:** 1 Anthony Nolan Research Institute and University College London, Royal Free Campus, Pond Street, London NW3 2QG, UK; 2 Institute of Child Health, 30 Guilford Street, London WC1N 1EH, UK

## Abstract

Graft versus Host Disease (GvHD) remains one of the main complications after hematopoietic stem cell transplantation (HSCT). Due to their ability to suppress effector cells, regulatory T cells (Tregs) have been proposed as a cellular therapy to prevent GvHD, however they also inhibit the functions of natural killer (NK) cells, key effectors of the Graft versus Leukemia effect. In this study, we have explored whether a Tregs therapy will also impact on NK cell differentiation. Using an *in vitro* model of hematopoietic stem cell (HSC) differentiation into NK cells, we found that activated Tregs led to a 90% reduction in NK cell numbers when added at the time of commitment to the NK cell lineage. This effect was contact dependent and was reversible upon Tregs depletion. The few NK cells that developed in these cultures were mature and exhibited normal functions. Furthermore, adoptive transfer of activated Tregs in rag^-/-^ γc^-/-^ mice abrogated HSC differentiation into NK cells thus confirming our *in vitro* findings. Collectively, these results demonstrate for the first time that activated Tregs can inhibit NK cell differentiation from HSC under specific conditions.

CD4^+^CD25^high^Foxp3^high^ regulatory T cells (Tregs) are involved in the maintenance of self-tolerance and immune homeostasis^1^. Tregs suppress a variety of immune cells such as T cells^2^,^3^, dendritic cells^4^, and natural killer (NK) cells^5^. Therefore, Tregs have been considered as an adoptive cell therapy to modulate Graft versus Host Disease (GvHD), one of the main complications after allogeneic hematopoietic stem cell transplantation (HSCT)^6^. Clinical studies suggest that the infusion of Tregs to prevent GvHD in transplanted patients is safe^7^,^8^,^9^,^10^, but the impact of Tregs on immune reconstitution still needs further investigation.

Tregs directly suppress the functions of targets via the action of immunosuppressive molecules such as transforming growth factor-β (TGF-β)^11^, interleukin (IL)-10^12^ or IL-35^13^, or by IL-2 deprivation in the milieu^14^. Studies in humans and mice demonstrated that Tregs inhibit NK cell functions via membrane bound TGF-β such as cytotoxicity and cytokine production^3^,^5^,^15^,^16^,^17^,^18^, decrease the expression of key activating receptors^5^,^15^, affect their proliferation^19^, and that Tregs depletion in mice leads to increased NK cell numbers^5^,^20^,^21^. It has also been demonstrated that Tregs regulate NK cells via IL-2 deprivation, limiting cytokine availability for NK cell activation and homeostasis^22^,^23^,^24^.

NK cells are immature in infants, leading to an increased susceptibility to infection^25^. The immaturity of infant and neonate NK cells has been linked to TGF-β expression^26^, with fetal NK cells being more susceptible to TGF-β than peripheral blood (PB) NK cells^27^. Moreover, TGF-β impacts hematopoietic stem cell (HSC) functions by skewing their differentiation towards the myeloid over the lymphoid lineage^28^. The overexpression of a key component of the TGF-β signaling cascade, SMAD4, in HSC from umbilical cord blood (CB) led to growth arrest and apoptosis of the transduced cells in response to TGF-β, and reduced reconstitution capacity of these cells *in vivo*^29^. Given that Tregs can control mature and immature NK cell responses, partly via TGF-β, and that TGF-β can affect HSC functions, one could expect that Tregs could also affect NK cell differentiation from HSC. Therefore, in the present study we assessed the effects of Tregs on HSC differentiation into NK cells *in vitro* and *in vivo*.

## Results

### Activated Tregs inhibit NK cell differentiation

**Figure 1:**
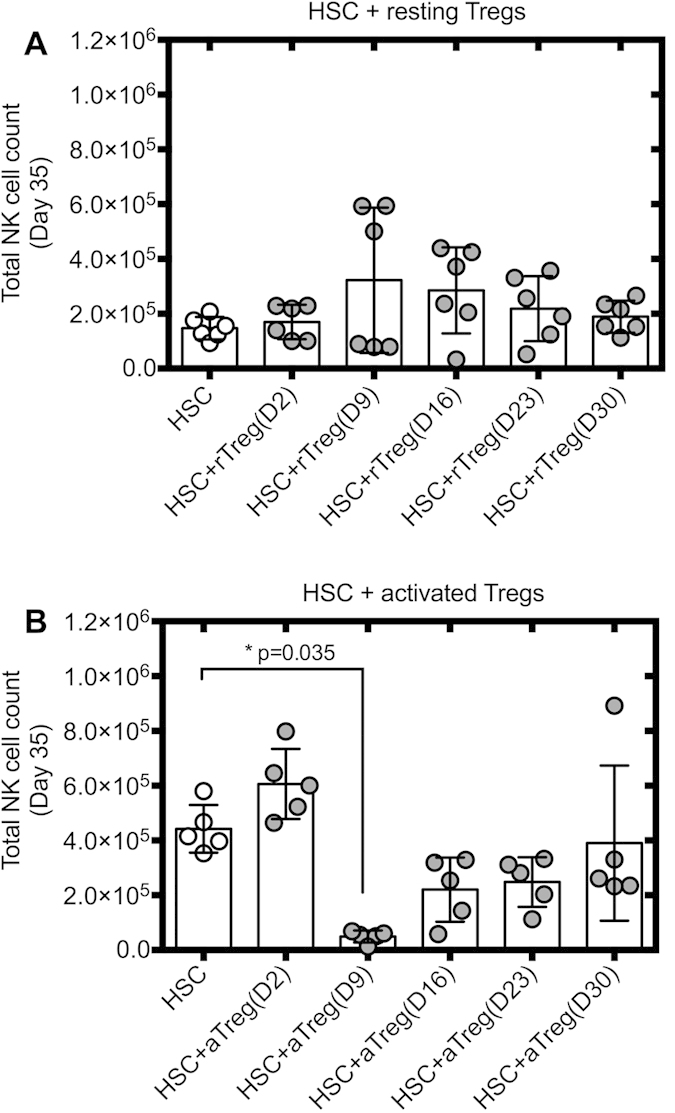
Activated Tregs, not resting Tregs, inhibit NK cell differentiation from HSC. HSC were cultured with resting or activated Tregs added at days 2, 9, 16, 23 and 30 of differentiation. (**A**) Total NK cell counts at day 35 of HSC cultures ± resting or (**B**) activated Tregs were assessed by flow cytometry (n = 5–6 per condition). Reported cell counts were calculated from total cell numbers and cell ratios were determined by flow cytometry. ***P ≤ 0.005.

To evaluate the effects of Tregs on NK cell differentiation, an established *in vitro* model of differentiation of CB HSC into NK cells was used^30^. This model is ideal to analyze the effect of Tregs on NK cell differentiation as HSC only differentiate into NK cells under the conditions used^31^. Allogeneic, resting or activated CB Tregs were added at key time points of HSC cultures (Figure S1). Numbers as well as percentages of NK cells and percentages of persisting Tregs were determined at day 35 of HSC cultures. Whilst resting Tregs did not affect HSC differentiation (Fig. 1A and Figure S2 for representative FACS plots), a significant reduction in NK cell numbers were observed when activated Tregs where added to HSC at day 9 but not at another time points (Fig. 1B and Figure S2), with 90% reduction in NK cell numbers observed. Viability and number of CD45^+^ cells in HSC cultures were not affected by the addition of Tregs (Figure S3).

### Activated Tregs block commitment to the NK cell lineage

**Figure 2:**
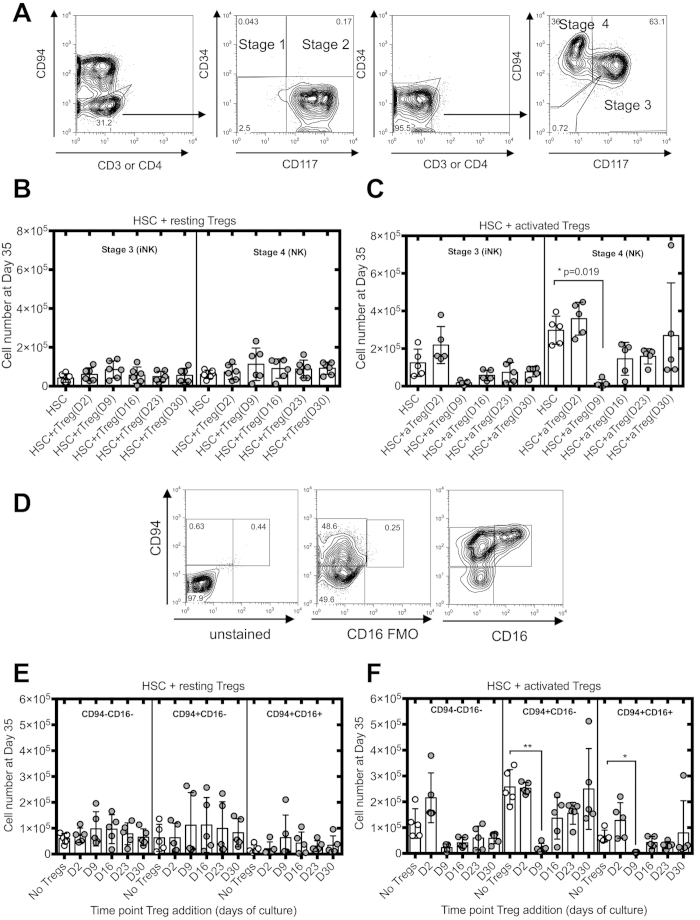
Activated Tregs decreased numbers of NK cells at different stages of differentiation and maturation. (**A**) Representative analysis of the different stages of NK cell differentiation. Total number of stages 3 and 4 of NK cell differentiation at day 35 of HSC cultures in the presence of resting (**B**) or activated Tregs (**C**) (n = 5). (**D**) Representative analysis of CD94 and CD16 expression by CD56^+^ NK cells at day 35 of HSC cultures. FMO represents fluorescence minus one and were used as controls to determine positivity of expression. Total numbers of NK cells in different stages of maturation at day 35 of HSC cultures in the presence of resting (**E**) or activated Tregs (**F**) (n = 5). Reported cell counts were calculated from total cell numbers and cell ratios were determined by flow cytometry. *P ≤ 0.05, **P ≤ 0.01.

We then assessed whether activated Tregs inhibited a specific stage of NK cell differentiation. To address this question, we used the differentiation model proposed by Freud and Caligiuri^32^, whereby the expression of CD34, CD117 and CD94 defined four stages of NK cell differentiation: pro-NK cells (stage 1) (CD3^−^CD34^+^CD117^−^CD94^−^), pre-NK cells (stage 2) (CD3^−^CD34^+^CD117^+^CD94^−^), committed immature (iNK) cells (stage 3) (CD3^−^CD34^−^CD117^+^CD94^−^) and CD56^bright^ NK cells (stage 4) (CD3^−^CD34^−^CD117^+/−^CD94^+^) (Fig. 2A). As Tregs seem to affect HSC differentiation only when added at day 9 and not at any other time point we characterized HSC cultures at each time point where Tregs were added in order to identify which cells are susceptible to Treg inhibition. We found that most of the cells are either in stage 1 or 2 of NK cell differentiation with a lower proportion being in stage 3 at day 7 of HSC culture (Figure S4). As the cultures progressed, cells were progressing to stage 4 with a low frequency of these cells at days 12 and 14 of culture. This frequency increased progressively to make up about 50% of the cells in cultures at later time points. This suggests that activated Tregs impact HSC differentiation by inhibiting either stages 1, 2 or potentially 3 of NK cell differentiation.

We then performed the same analysis at day 35 in HSC cultures in which resting or activated Tregs were added. Data are not shown for stage 1 and 2 cells due to low cell numbers at the end of HSC cultures, as all cells generated are NK cells. When resting Tregs were added to HSC cultures, no difference was detected in total cell numbers of NK cell stages 3 and 4 (Fig. 2B). However, when activated Tregs were added at day 9 of culture, the numbers of cells in stage 3 and 4 were reduced by 90% (Fig. 2C).

Freud and Caligiuri reported a fifth stage of NK cell differentiation^32^. Three different populations can be defined according to CD94 and CD16 expression: CD56^+^CD94^−^CD16^−^, CD56^+^CD94^+^CD16^−^ and CD56^+^CD94^+^CD16^+^ (Fig. 2D), the latest being the most mature NK cell population. While resting Tregs had no effect on the number of cells in the last differentiation stages, a significant reduction in cell number of CD56^+^CD94^+^CD16^−^ and CD56^+^CD94^+^CD16^+^ was noted when activated Tregs were added at day 9 of HSC cultures (Fig. 2E,F respectively).

### NK cells differentiated in the presence of activated Tregs are fully functional

**Figure 3:**
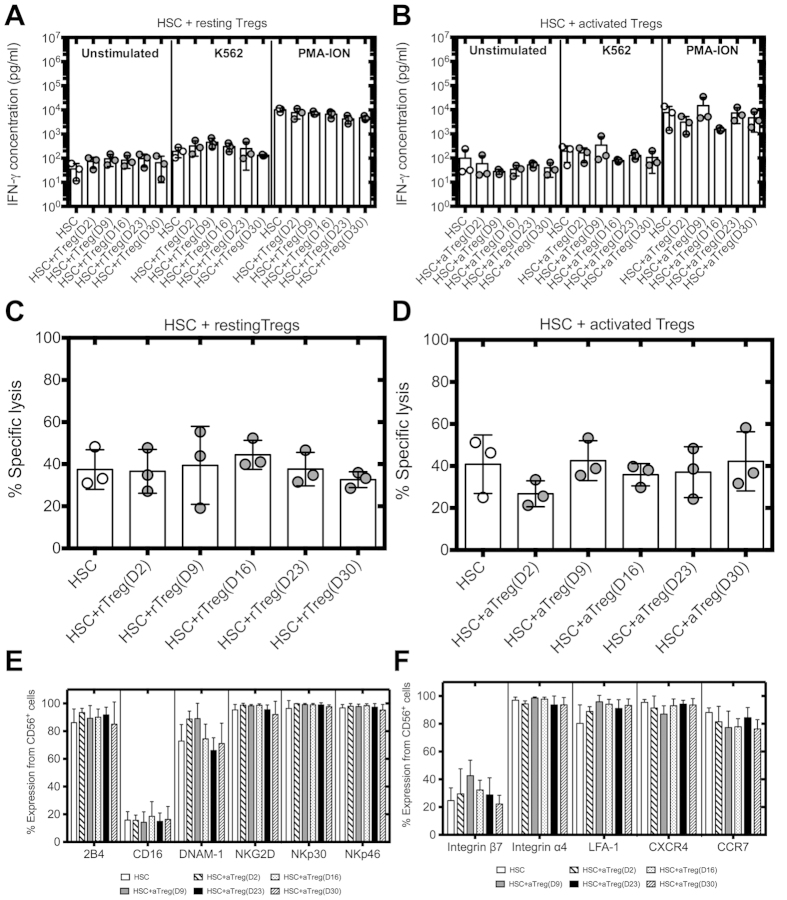
Activated Tregs have no effect on the repertoire and function of fully differentiated NK cells. IFN-γ secretion of differentiated NK cells ± resting (**A**) or activated Tregs **(B**) added at different time points of HSC cultures (n = 3). NK cells harvested at day 35 of HSC cultures were stimulated with or without K562 cells at a ratio of 1:1 or PMA-ION for 2 h. Supernatants were collected and analyzed by ELISA. Cytotoxicity of differentiated NK cells ± resting (**C**) or activated Tregs (**D**) added at different time points of HSC cultures. NK cells from day 35 of HSC cultures were incubated with K562 cells at a ratio of 1:5 and cytotoxicity analyzed by Cr^51^ release assay (n = 3). (**E**) Expression of activating receptors on NK cells from day 35 of HSC cultures ± activated Tregs. (**F**) Expression of chemokine receptors and integrins by NK cells from day 35 of HSC cultures ± activated Tregs (n = 3, 6).

To investigate whether the few cells that acquired a mature NK cell phenotype in the presence of activated Tregs were functional, we tested whether these NK cells could lyse K562 cells *in vitro* and produce IFN-γ. We found that resting and activated Tregs had no significant effect on IFN-γ secretion (Fig. 3A,B respectively) or killing of K562 by the differentiated NK cells (Fig. 3C,D respectively). The addition of activated Tregs to HSC cultures had no impact on the expression of 2B4, CD16, DNAM-1, NKG2D, NKp30 and NKp46 by the differentiated NK cells (Fig. 3E), key receptors involved in NK cell mediated cytotoxicity. Moreover, NK cells differentiated in presence of Tregs exibited normal expression of trafficking receptors (Fig. 3F).

### Tregs-mediated inhibition of NK cell differentiation is cell contact dependent and is reduced following Tregs depletion

**Figure 4:**
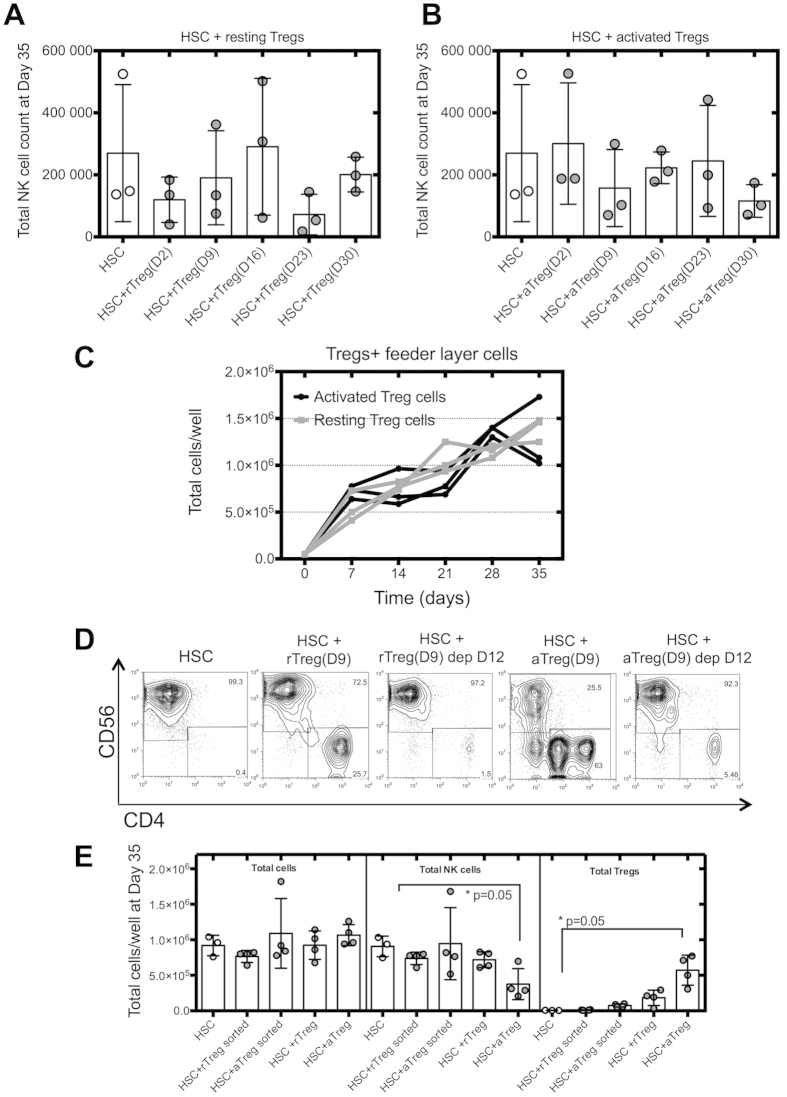
Tregs-mediated regulatory function is cell contact dependent and cytokine competition independent. HSC were cultured with resting (**A**) or activated Tregs (**B**) added at different time points of HSC cultures. Cells were separated using Transwell to assess cell contact dependency of the Treg effect. Cells were analyzed by cell count and flow cytometry at day 35 of HSC cultures (n = 3). (**C**) Resting or activated Tregs were cultured without HSC using NK cell differentiation culture conditions. Treg cell numbers were evaluated at different time points (n = 3). (**D**) Resting or activated Tregs were added at day 9 of HSC cultures and depleted at day 12 by cell sorting. HSC depleted (dep) of Tregs were cultured with freshly irradiated feeder layer cells and cytokines. Representative flow cytometric analysis of CD56 vs CD4 HSC cultures, depleted or not of Treg cells by cell sorting. (**E**) Total lymphocyte, NK cell and Treg cell counts at day 35 of HSC cultures (n = 4). Reported cell counts were calculated from total cell numbers and cell ratios were determined by flow cytometry.

Tregs affect NK cell functions in a TGF-β, cell contact dependent manner^5^,^16^,^33^, or by limiting IL-2 availability^22^,^23^. Therefore, we evaluated whether direct cell contact was necessary for activated Tregs to affect NK cell differentiation. We performed transwell cell cultures between HSC and Tregs, and analyzed NK cell numbers at day 35 of culture. Resting Tregs had no effect on NK cell differentiation from either transwell or regular HSC cultures (Fig. 4A). However, the inhibition of NK cell differentiation mediated by activated Tregs when added at day 9 of HSC cultures was reduced when HSC and activated Tregs were separated by a transwell (Fig. 4B), highlighting the requirement for cell contact for activated Tregs to exert their suppressive effect on NK cell differentiation.

We then studied whether Tregs could suppress NK cell differentiation by cytokine consumption in our *in vitro* system. Tregs were cultured without HSC but using the culture conditions that support HSC differentiation into NK cells. If activated Tregs inhibit NK cell differentiation from HSC via cytokine consumption, one would expect activated Tregs to exhibit a different proliferative profile than resting Tregs. However, we did not observe a difference in proliferative rates between resting and activated Tregs over the 35 days of culture (Fig. 4C), suggesting that activated Tregs do not inhibit NK cell differentiation by depleting the milieu of cytokines.

As Tregs persist in HSC cultures over the period studied (Figure S5), we assessed whether their depletion could restore HSC differentiation into NK cells. Tregs were added at day 9 of HSC cultures and depleted by cell sorting at day 12. HSC were then re-cultured as previously described and NK cell numbers analyzed at day 35 of culture. When activated Tregs were depleted, NK cell differentiation from HSC could be restored reaching equivalent numbers of NK cells and fold expansion rates as in control HSC cultures (Fig. 4D,E), showing that Tregs persistence is required for inhibition of NK cell differentiation to be maintained.

### Recombinant TGF-β blocks NK cell differentiation and recapitulates the effects of activated Tregs on HSC

**Figure 5:**
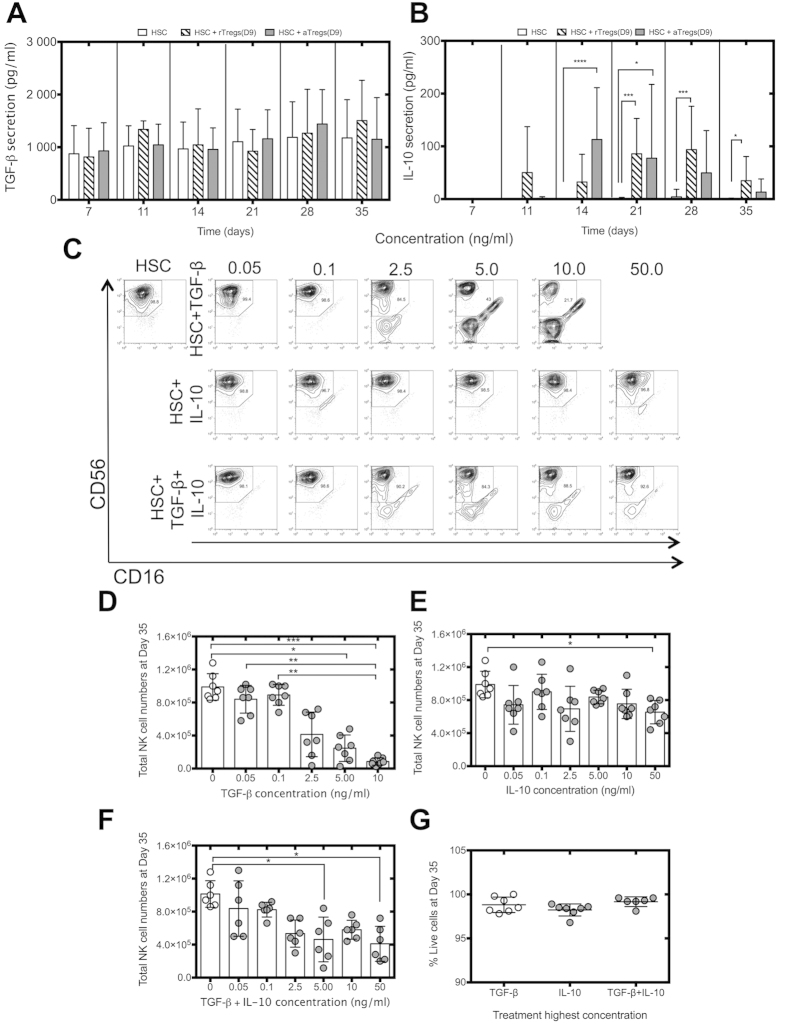
TGF-β inhibits NK cell differentiation. HSC were cultured ± resting (**A**) or activated Tregs (**B**) added at day 9 of HSC cultures at a 1:4 ratio (Tregs:HSC). Supernatants were collected every week and TGF-β and IL-10 secretion were analyzed by ELISA (n = 6–9). (**C**) Recombinant human TGF-β and IL-10 were added at days 9, 14, 21 and 28 of HSC cultures. Representative flow cytometric analysis of CD56 vs CD16 of HSC cultures treated with recombinant TGF-β and IL-10 are shown. NK cell counts of HSC cultures treated with recombinant TGF-β (**D**), IL-10 (**E**) or both molecules (**F**) (n = 6–7). (**G**) Viability of HSC cultures treated with recombinant TGF-β and IL-10, day 35 (n = 7). Reported cell counts were calculated from total cell numbers and cell ratios were determined by flow cytometry. *P ≤ 0.05, **P ≤ 0.01, ***P ≤ 0.001.

In order to elucidate how activated Tregs inhibit NK cell differentiation from HSC, we assessed the levels of TGF-β and IL-10 secretion in HSC cultures, as both are key molecules in Tregs-mediated suppression^5^,^11^,^12^. High levels of TGF-β were found in all cultures regardless of the addition of resting or activated Tregs with no significant differences observed (Fig. 5A), suggesting that TGF-β is secreted during cultures of CD34+ cells under the conditions used. IL-10 was detected at different time points in the supernatant of HSC cultures when either resting or activated Tregs were added (Fig. 5B). We then assessed whether blocking these molecules prevented the Tregs-mediated inhibition of NK cell differentiation observed. The blocking agents used were SB 431542, a specific inhibitor of the TGF-β receptor kinase and a blocking antibody against the IL-10 receptor α. However, these reagents affected the viability of HSC cultures and did not allow this strategy to be pursued. Therefore, we assessed the effects of the addition of these molecules on HSC cultures by adding recombinant human TGF-β or IL-10 at day 9 of HSC culture and then weekly to mimic persistence of Tregs and to study whether the addition of these molecules to HSC cultures could affect NK cell differentiation in a similar manner as activated Tregs (Fig. 5C). Cytokine concentrations were chosen based on secretion levels observed in this study and those previously published by others^5^,^29^,^34^. Only concentrations between 5 to 10 ng/ml of TGF-β (Fig. 5D) and the highest concentration of recombinant IL-10 tested impacted on NK cell differentiation (Fig. 5E). Similarly, the addition of both TGF-β and IL-10 inhibited NK cell differentiation from HSC at the highest concentrations tested (Fig. 5F). Cell viability was not compromised by the addition of either recombinant TGF-β or IL-10 (Fig. 5G). Taking together with the importance of cell-to-cell contact for activated Tregs to suppress in this system, this suggests that activated Tregs may suppress HSC differentiation into NK cells via membrane bound and not secreted TGF-β.

### Activated Tregs decreased expression levels of mRNAs that regulate NK cell maturation and affect cell proliferation

**Figure 6:**
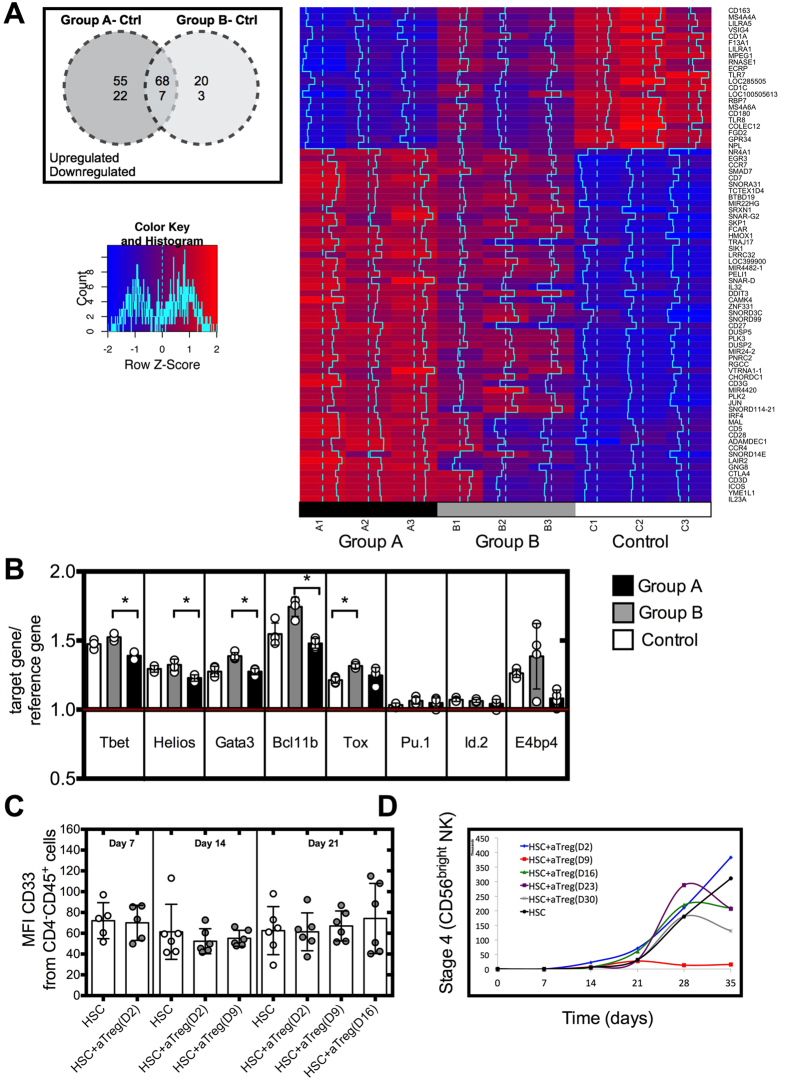
Molecular analysis and proliferation of HSC cultured with resting or activated Tregs. (**A**) Activated Treg cells (Group A) or Resting Tregs (Group B) were added at day 9 of HSC cultures. HSC were then isolated by cell sorting at day 12 of culture, RNA was extracted and analyzed by microarray analysis (n = 3) or (**B**) by real time PCR (n = 4) HSC cultures were used as controls. (**C**) Analysis of CD33 expression at different time points of HSC cultured with activated Tregs (n = 5–6). (**D**) Total cell number of HSC cultures in the presence of activated (Tregs n = 5). Reported cell counts were calculated from total cell numbers and cell ratios were determined by flow cytometry. *P ≤ 0.05.

To identify molecular changes underlying the effects of Tregs on NK cell differentiation, we performed a microarray analysis on HSC cultured with resting or activated Tregs added at day 9 of culture and isolated by cell sorting at day 12 of culture. There were clear transcriptional changes in HSC cultured between resting or activated Tregs compared to HSC cultured alone. Using a cut-off of log2fold change (logFC) expression and adjusted p value < 0.05, we found a total of 55 upregulated and 22 downregulated genes that were exclusively differentially regulated when activated Tregs (group A) were added to HSC cultures while 20 genes were upregulated and 3 down-regulated when resting Tregs (group B) were added to HSC cultures (Fig. 6A). A summary of the genes of interest is presented in Fig. 6A and Table S2.

As compared to resting Tregs, activated Tregs present in HSC cultures induced lower levels of Tox (logFC of 0.54 and 0.76 respectively) and higher levels of Bcl11b (logFC of 1.40 and 0.54 respectively), important transcription factors for NK cell differentiation and maturation but yet expressed similar levels when compared to the control group (HSC alone). Furthermore, HSC cultured with resting or activated Tregs exhibited similar mRNA levels of E4bp4, key transcription factor involved in NK cell differentiation, and of Gata-3 and T-bet, relevant for NK cell maturation (Table S2). To confirm these findings, we performed a real time PCR analysis and found that activated Tregs did not significantly alter mRNA levels of Bcl11b, E4bp4, Gata-3, Id.2, Helios, Pu.1, T-bet, Tox in HSC (Fig. 6B), thus suggesting that activated Tregs do not inhibit HSC differentiation into NK cells by impacting on the expression of transcription factors involved in NK cell differentiation.

TGF-β can skew differentiation of HSC towards the myeloid lineage over the lymphoid lineage^28^. The microarray analysis did not highlight differences in expression of factors involved in differentiation towards one lineage over another. Moreover, we did not detect any change in protein expression of the myeloid marker, CD33, in the different cultures analyzed when Tregs were added, as assessed by flow cytometry (Fig. 6C). No expression of CD14 was detected at any time point of HSC culture (data not shown). Overexpression of SMAD4, a key component of the TGF-β signaling cascade in CB HSC, inhibited cell proliferation of the transduced cells in response to TGF-β^29^. Notably, our microarray analysis revealed downregulation of genes involved in cell division such as cell division cycle 23 (FC of −0.45, p = 0.034), cell division cycle associated 7-like (FC of −1.028 p = 0.036) cell death-inducing DFFA-like effector b (FC of −0.91 p = 0.001) and growth arrest-specific 6 (FC of −0.70 p = 0.002) in HSC cultured with activated Tregs (Table S2). Furthermore, the gene regulator of cell cycle was consistently upregulated (logFC of 2.42 p < 0.001) in HSC cultured with activated Tregs. After the addition of activated Tregs at day 9 of culture, no cell proliferation was observed, as cell numbers were constant in these HSC cultures from that time point onwards (Fig. 6D), suggesting a possible block in cell proliferation of HSC cultured with activated Tregs.

### Tregs impair NK cell differentiation *in vivo*

**Figure 7:**
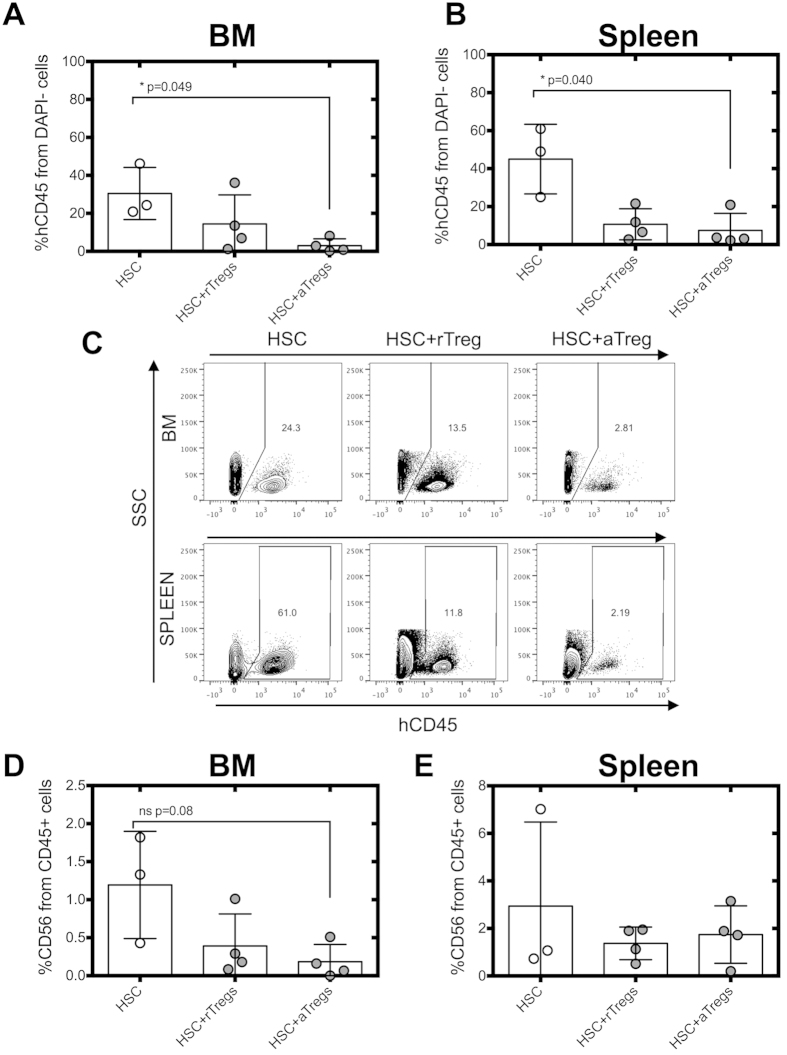
Adoptive transfer of Tregs in rag^−/−^γc^−/−^ mice transplanted with HSC. Rag^−/−^γc^−/−^ mice were injected with 2 × 10^5^ freshly isolated CB CD34^+^ HSC ± allogeneic resting or activated Tregs at a 1:1 ratio and analyzed after ten weeks. BM and spleen from rag^−/−^γc^−/−^ mice were assessed for human NK cell reconstitution (human CD45; hCD45) by flow cytometry. Frequency of expression of hCD45^+^ cells gated on live cells (DAPI^−^) of BM (**A**) and spleen (**B**). (**C**) Representative flow cytometric analysis of hCD45 vs SSC of BM and spleen tissues. Frequency of expression of CD56 NK cells within hCD45^+^ live cells of BM (**D**) and spleen (**E**). Data is representative of 3–4 mice per group. aTreg: activated Tregs, rTreg: resting Tregs.

To confirm the *in vitro* data, we determined whether Tregs were able to inhibit HSC differentiation *in vivo* using a previously described humanized mouse model of NK cell differentiation^35^, which has been used to analyze the effect of Tregs on the immune response against various tumors^36^. Because of the low NK cell numbers obtained in the mouse model, we were unable to measure the time point at which cells commit to the NK cell lineage. Therefore, we decided to infuse resting or activated Tregs together with HSC to ensure that Tregs were present before and at the time of NK cell commitment. Resting or activated Tregs were infused together with HSC into the liver of irradiated 3–5 days old Rag^−/−^γc^−/−^ mice. The spleen and bone marrow (BM) of the mice were analyzed 10 weeks after injection for the presence of human CD45^+^ leukocytes and NK cells. We found a lower percentage of hCD45^+^ cells in the BM and the spleen of the mice injected with HSC and activated Tregs (Fig. 7A–C). A reduction in the percentage of NK cells in the BM of mice infused with HSC and activated Tregs was also observed, while no difference was observed in the spleen (Fig. 7C). CD4^+^ T cells did not persist in the BM or spleen of the mice injected with resting or activated Tregs and no T cells or NKT cells were detected in the BM or the spleen (data not shown).

## Discussion

Several authors have demonstrated that Tregs can abrogate NK cell functions under certain conditions^3^,^5^,^15^,^17^,^18^,^19^. It has been suggested that Tregs act as facilitators of allogeneic tolerance^37^ and might help HSC engraftment^38^. Here we demonstrated for the first time that activated Tregs impair NK cell differentiation *in vitro and in vivo.* We found approximately 90% reduction in NK cell count when activated Tregs were added to HSC cultures at the time point when commitment to the NK cell lineage occurs, whereas no effect was observed when resting Tregs were added. Cell numbers for the final stages of NK cell differentiation were severely compromised in HSC cultures with activated Tregs. Importantly, co-infusion of HSC and activated Tregs in a humanized model of transplantation confirms that Tregs are able to suppress NK cell differentiation *in vivo*. Remarkably, the few cells that acquire a NK cell phenotype in the presence of activated Tregs *in vitro* were functional and exhibited normal IFN-γ production and cytotoxicity. Tregs did not affect the expression of any of the activating receptors analyzed on the differentiated NK cells. These results were unexpected since it has been described that TGF-β induced downregulation of NKG2D and NKp30 by NK cells^39^. However, these latest data were obtained using isolated NK cells and not NK cells differentiated from HSC, which could explain why in our study Tregs did not affect NK cell functions and phenotype.

The addition of recombinant TGF-β to HSC cultures, but not IL-10, induced similar reduction in NK cell counts as when activated Tregs were added to HSC cultures. A similar effect has also been observed in mice by Marcoe and colleagues who demonstrated that TGF-β can also affect NK cell differentiation, with TGF-β being responsible for the immaturity of NK cells in this model^26^. Some authors have also suggested a dual effect of TGF-β on HSC differentiation with suppressive effects at high concentrations and stimulatory effects at low concentrations:^40^,^41^ yet this effect was not observed in our *in vitro* system.

Tregs have previously been shown to suppress NK cell functions in a cell contact dependent manner^5^ and their depletion led to recovery of NK cell effector functions^16^. We showed that cell contact between activated Tregs and HSC was required for Tregs-mediated inhibition of NK cell differentiation, since physical separation of activated Tregs from HSC abrogated the effect. It was expected that transcription factors involved in NK cell differentiation would be differentially expressed in HSC in the presence of activated Tregs. Studies in mice highlight the importance of E4bp4, Pu.1, Id.2 and Tox in NK cell differentiation, since mice deficient in these transcription factors have reduced NK cell numbers^42^,^43^,^44^,^45^. However, our gene profiling analysis showed that HSC cultured with activated Tregs exhibited normal transcription of genes involved in NK cell differentiation and maturation. In addition, activated Tregs did not favor HSC differentiation towards another lineage in our system. In fact, we observed a constant cell number in HSC cultures with activated Tregs and our gene profiling analysis highlighted changes in expression of genes that regulate cell cycle suggesting that activated Tregs might block proliferation of HSC in our system.

Nguyen and colleagues^47^ used a mouse model of GvHD with mismatched HSC and adoptive transfer of effector T cells and Tregs and showed enhanced NK cell reconstitution and improved viral clearance in comparison to mice injected with effector T cells only^46^,^47^. As clinical trials that assessed the use of Tregs as a therapy for GvHD focused on clinical safety, limited data on the effects of Tregs on immune reconstitution have been reported. For instance, Brunstein and colleagues assessed whether expanded CB Treg cells could prevent GvHD in double CBT patients and provide some evidence that Treg cells may impair immunity to infections^7^. In this study, higher susceptibility to early viral reactivation was observed within 30 days after transplantation in Tregs treated patients as compared to historical controls^7^. However, Tregs could be detected for a maximum of two weeks in these patients, so Tregs might not have persisted for long enough and in sufficient numbers to impact on immune reconstitution in this study. Di Ianni and colleagues demonstrated that adoptive transfer of freshly isolated donor Tregs counteracted the potential GvHD induced by megadoses of donor Tcon cells in patients receiving a haploidentical graft^8^. When compared to a cohort of 152 patients, Tregs patients exhibited higher immune reconstitution and improved immunity to opportunistic infections, thus implying that under these particular conditions, Tregs did not impair NK cell immune reconstitution. This could be because of the different type of conditioning used as well as the high HSC numbers infused and their ability to override a potential Tregs-mediated effect. In preclinical studies, it is not known when HSC commit to the NK cell lineage *in vivo*, but it is likely that this process would occur within the first weeks post-transplantation. More studies are warranted to assess the impact of a Treg therapy in the context of HSCT on GvHD but also on other parameters such as immune reconstitution.

The effects that Tregs have on NK cell differentiation may have critical implications in the clinic as they could severely compromise NK cell reconstitution. In conclusion, this study may therefore suggest possible conditions under which Tregs inhibit NK cell differentiation, thus providing valuable information for the future design of Tregs-based therapies.

## Material and Methods

### CB samples and cell lines

All CB samples were obtained with prior written consent and ethical committee approval from the Anthony Nolan Cord Blood bank (Research Ethics Committee reference 10/H0405/27). The study had full ethical approval from the Anthony Nolan and Royal Free Hospital Research Ethics Committee. K562 cells were cultured in RPMI with 10% FBS. EL08.1D2 cells were cultured as previously described^30^.

### HSC differentiation into NK cells

CB mononuclear cells were prepared by density centrifugation using Ficoll-Paque^TM^ premium (GE Healthcare) and HSC were isolated using the CD34 MicroBead kit^48^ and frozen for future use. The purity of CD34+ cells ranged from 90 to 98%. Thawed CD34^+^ cells were plated on irradiated EL08.1D2 cells and cultured as described by Grzywacz *et al.*^30^ except for the addition of 50 ng/mL IL-15 for the last two weeks^31^. Where indicated, different concentrations of human recombinant TGF-β (R&D Systems) and IL-10 (Prospec) were added.

### Tregs isolation and stimulation

CB Tregs were isolated after ficoll using the CD4^+^CD25^+^ Regulatory T cell isolation kit (Miltenyi Biotec)^49^. Purity ranged from 90 to 96%. For TCR stimulation, plates were coated for 2 h at 37 °C with 10 μg/mL anti-CD3 (HIT3a, BD Biosciences) and Tregs were cultured with 10 μg/mL anti-CD28 (CD28.2, BD Biosciences) and 1000 IU/mL IL-2 (Prospec) for 24 h at 37 °C. Activation was assessed by analyzing CD69, CTLA4, GITR and LAP expression by flow cytometry (Figure S6). Tregs were washed and added to HSC cultures at a 1:4 ratio, except from day 2 in which a 1:1 ratio was used. For transwell cultures, Tregs were placed in the upper chamber of HTC Transwell plates (Corning). The bottom chamber was coated with irradiated EL08.1D2 cells and HSC added as previously described.

### Cell sorting

HSC were cultured with Tregs from day 9 until day 12, and then Tregs and HSC were re-isolated using a MoFlow cell sorter (Beckman Coulter) by sorting CD4^−^ and CD4^+^ cell populations (95–99% purity). HSC were then re-cultured with freshly irradiated EL08.1D2 feeder layer cells and cytokines.

### Flow cytometry

The following monoclonal antibodies were purchased from BD Biosciences: 7-AAD, CCR7, CD3, CD4, CD16, CD14, CD19, CD25, CD34, CD45, CD56, CD69, CD94, CD117, CTLA4 and DAPI; from eBiosciences: CD62L and CD127; from R&D Systems: CCR5, CCR6, CXCR1, CXCR3, GITR, integrin β7 and LAP. Titrated amounts of 7AAD and DAPI were added to stained cells 10 minutes before acquisition. For Foxp3 intranuclear staining, cells were then fixed and permeabilized at room temperature for 15 minutes using the Foxp3 Fix/Perm Kit (eBioscience) and stained in permeabilization buffer with a directly labeled antibody to Foxp3 APC (PCH101; eBioscience) at room temperature for 45 minutes, prior to two washes with permeabilization buffer. The LSRFortessa and FACSCalibur (Becton Dickinson) cell analyzers were used to acquire data. FlowJo software (Tree Star) was used for data analysis.

### Cytotoxicity assays

K562 cells were labeled for 45 min with 100 μCi/1 × 10^6^ cells at 37 °C using ^51^Chromium (^51^Cr). After 4 h co-culture with NK cells, supernatants were collected to measure ^51^Cr release.

### ELISA assays

IFN-γ, IL-10 and TGF-β Ready-SET-Go! ELISA kits (eBioscience) were used according to the manufacturer’s instructions. 2 × 10^5^ NK cells were either simulated with 100 ng/mL phorbol 12-myristate 13-acetate and 1 μg/mL Ionomycin (PMA-ION) or with K562 cells at a 1:1 ratio and incubated for 4 h.

### Real time PCR

RNA extraction was performed using the RNeasy mini kit (Qiagen). cDNA was prepared using the Superscript III Reverse Transcriptase (Life Technologies) and Random Primers (Promega). PCR reactions were carried out using SYBRgreen (PrimerDesign). The housekeeping genes used were ATP5B, UBC and TOP1 (PrimerDesign). Primer sequences are described in Table S1. Results are presented as relative expression. The higher the ratio, the lower the amount of mRNA of the target gene.

### Microarray analysis

RNA samples were extracted using the RNeasy mini kit (Qiagen) and processed using GeneChip Whole Transcript Sense Target Labeling Assays with the Ambion WT Expression kit and Affymetrix GeneChip WT Terminal Labeling and Controls kit (Affymetrix). The resulting ssDNAs were hybridized to GeneChip human Gene 2.0 ST Arrays (Affymetrix). The microarray analysis was performed by the UCL genomics facility, Wolfson Institute for Biomedical Research. Initial reads were processed through Affymetrix software to obtain raw .cel files. Microarray data were background-corrected and normalized using the robust multi-array average (RMA) algorithm. Fold change expression values were calculated by LIMMA (R software) as the log2 fold change of the signal values of the experimental groups (HSC with activated or resting Tregs) compared with the control group (HSC only). Gene expression changes with ≥2 log2 fold alterations and adjusted p values ≤0.05 were considered significant. The Benjamini and Hochberg’s method was used to adjust p-values. Heatmap generator software was used to present the data^50^. Microarray data are available from the GEO repository (GSE55091).

### *In vivo* model of NK cell differentiation

Rag^−/−^γc^−/−^ mice (3–5 days old) received sublethal irradiation and were injected intra-hepatically with 2 × 10^5^ human CB CD34^+^ HSC only or an equal number of Tregs. Between week 1 and 3, mice were injected i.p. weekly with hIL-15 (15 ng; R&D Systems), hIL-7 (30 ng, Prospec), hSCF (30 ng; Prospec), hFLT-3 (15 ng; Prospec), and hIL-3 (75 ng; only week 1–2; R&D Systems). hIL-15 (75 ng) was added between week 4–5. hIL-15 (2.5 μg) and hIL-15Rα-Fc (7.5 μg; R&D Systems) between weeks 6–9. NK cell differentiation was then analyzed in the spleen and BM of the mice 10 weeks after injection. Animal experiments were performed according to the recommendations of the UK Home Office Regulations, protocols were approved by the UK Home Office (project license 80/2456).

### Statistical analysis

All analysis was performed with PRISM v.6 (GraphPad). When three or more groups were compared, a non-parametric Friedman’s (for *in vitro* data) or Kruskal-Wallis (for *in vivo* data) test with Dunn’s multiple comparison was applied. When two groups were analyzed, Mann-Whitney test was performed.

## Additional Information

**How to cite this article**: Pedroza-Pacheco, I. *et al.* Regulatory T cells inhibit CD34+ cell differentiation into NK cells by blocking their proliferation. *Sci. Rep.* **6**, 22097; doi: 10.1038/srep22097 (2016).

## Supplementary Material

Supplementary Information

## References

[b1] Miyara M. & Sakaguchi S. Human FoxP3(+)CD4(+) regulatory T cells: their knowns and unknowns. Immunol Cell Biol 89, 346–351 (2011). 21301480 10.1038/icb.2010.137

[b2] Thornton A. M. & Shevach E. M. Suppressor effector function of CD4+CD25+ immunoregulatory T cells is antigen nonspecific. Journal of immunology 164, 183–190 (2000). 10.4049/jimmunol.164.1.183 10605010

[b3] Trzonkowski P., Szmit E., Myśliwska J., Dobyszuk A. & Myśliwski A. CD4+CD25+ T regulatory cells inhibit cytotoxic activity of T CD8+ and NK lymphocytes in the direct cell-to-cell interaction. Clinical Immunology 112, 258–267 (2004). 15308119 10.1016/j.clim.2004.04.003

[b4] Fallarino F. *et al.* Modulation of tryptophan catabolism by regulatory T cells. Nature immunology 4, 1206–1212 (2003). 14578884 10.1038/ni1003

[b5] Ghiringhelli F. *et al.* CD4+CD25+ regulatory T cells inhibit natural killer cell functions in a transforming growth factor-beta-dependent manner. The Journal of experimental medicine 202, 1075–1085 (2005). 16230475 10.1084/jem.20051511 PMC2213209

[b6] Ferrara J. L., Levine J. E., Reddy P. & Holler E. Graft-versus-host disease. Lancet 373, 1550–1561 (2009). 19282026 10.1016/S0140-6736(09)60237-3 PMC2735047

[b7] Brunstein C. G. *et al.* Infusion of *ex vivo* expanded T regulatory cells in adults transplanted with umbilical cord blood: safety profile and detection kinetics. Blood 117, 1061–1070 (2011). 20952687 10.1182/blood-2010-07-293795 PMC3035067

[b8] Di Ianni M. *et al.* Tregs prevent GVHD and promote immune reconstitution in HLA-haploidentical transplantation. Blood 117, 3921–3928 (2011). 21292771 10.1182/blood-2010-10-311894

[b9] Edinger M. & Hoffmann P. Regulatory T cells in stem cell transplantation: strategies and first clinical experiences. Curr Opin Immunol 23, 679–684 (2011). 21802270 10.1016/j.coi.2011.06.006

[b10] Trzonkowski P. *et al.* First-in-man clinical results of the treatment of patients with graft versus host disease with human *ex vivo* expanded CD4+CD25+CD127- T regulatory cells. Clinical immunology 133, 22–26 (2009). 19559653 10.1016/j.clim.2009.06.001

[b11] Nakamura K., Kitani A. & Strober W. Cell contact-dependent immunosuppression by CD4(+)CD25(+) regulatory T cells is mediated by cell surface-bound transforming growth factor beta. J Exp Med 194, 629–644 (2001). 11535631 10.1084/jem.194.5.629 PMC2195935

[b12] Annacker O. *et al.* CD25+ CD4+ T cells regulate the expansion of peripheral CD4 T cells through the production of IL-10. Journal of immunology 166, 3008–3018 (2001). 10.4049/jimmunol.166.5.3008 11207250

[b13] Collison L. W. *et al.* The inhibitory cytokine IL-35 contributes to regulatory T-cell function. Nature 450, 566–569 (2007). 18033300 10.1038/nature06306

[b14] Pandiyan P., Zheng L., Ishihara S., Reed J. & Lenardo M. J. CD4+CD25+Foxp3+ regulatory T cells induce cytokine deprivation-mediated apoptosis of effector CD4+ T cells. Nature immunology 8, 1353–1362 (2007). 17982458 10.1038/ni1536

[b15] Bergmann C. *et al.* Human tumor-induced and naturally occurring Treg cells differentially affect NK cells activated by either IL-2 or target cells. European journal of immunology 41, 3564–3573 (2011). 21905023 10.1002/eji.201141532

[b16] Smyth M. J. *et al.* CD4+CD25+ T regulatory cells suppress NK cell-mediated immunotherapy of cancer. J Immunol 176, 1582–1587 (2006). 16424187 10.4049/jimmunol.176.3.1582

[b17] Zhou H., Chen L., You Y., Zou L. & Zou P. Foxp3-transduced polyclonal regulatory T cells suppress NK cell functions in a TGF-β dependent manner. Autoimmunity 43, 299–307 (2010). 20166879 10.3109/08916930903405875

[b18] Sun X. *et al.* CD39/ENTPD1 expression by CD4+Foxp3+ regulatory T cells promotes hepatic metastatic tumor growth in mice. Gastroenterology 139, 1030–1040 (2010). 20546740 10.1053/j.gastro.2010.05.007 PMC2930043

[b19] Romagnani C. *et al.* Activation of human NK cells by plasmacytoid dendritic cells and its modulation by CD4+ T helper cells and CD4+ CD25hi T regulatory cells. Eur J Immunol 35, 2452–2458 (2005). 15997468 10.1002/eji.200526069

[b20] Kim J. M., Rasmussen J. P. & Rudensky A. Y. Regulatory T cells prevent catastrophic autoimmunity throughout the lifespan of mice. Nature immunology 8, 191–197 (2007). 17136045 10.1038/ni1428

[b21] Feuerer M., Shen Y., Littman D. R., Benoist C. & Mathis D. How punctual ablation of regulatory T cells unleashes an autoimmune lesion within the pancreatic islets. Immunity 31, 654–664 (2009). 19818653 10.1016/j.immuni.2009.08.023 PMC2998796

[b22] Sitrin J., Ring A., Garcia K. C., Benoist C. & Mathis D. Regulatory T cells control NK cells in an insulitic lesion by depriving them of IL-2. The Journal of experimental medicine 210, 1153–1165 (2013). 23650440 10.1084/jem.20122248 PMC3674700

[b23] Gasteiger G. *et al.* IL-2-dependent tuning of NK cell sensitivity for target cells is controlled by regulatory T cells. The Journal of experimental medicine 210, 1167–1178 (2013). 23650441 10.1084/jem.20122462 PMC3674692

[b24] Gasteiger G., Hemmers S., Bos P. D., Sun J. C. & Rudensky A. Y. IL-2-dependent adaptive control of NK cell homeostasis. The Journal of experimental medicine 210, 1179–1187 (2013). 23650439 10.1084/jem.20122571 PMC3674698

[b25] Guilmot A., Hermann E., Braud V. M., Carlier Y. & Truyens C. Natural killer cell responses to infections in early life. J Innate Immun 3, 280–288 (2011). 21411972 10.1159/000323934

[b26] Marcoe J. P. *et al.* TGF-beta is responsible for NK cell immaturity during ontogeny and increased susceptibility to infection during mouse infancy. Nature immunology 13, 843–850 (2012). 22863752 10.1038/ni.2388 PMC3426626

[b27] Ivarsson M. A. *et al.* Differentiation and functional regulation of human fetal NK cells. J Clin Invest 123, 3889–3901 (2013). 23945237 10.1172/JCI68989 PMC3754261

[b28] Challen G. A., Boles N. C., Chambers S. M. & Goodell M. A. Distinct hematopoietic stem cell subtypes are differentially regulated by TGF-beta1. Cell Stem Cell 6, 265–278 (2010). 20207229 10.1016/j.stem.2010.02.002 PMC2837284

[b29] Rorby E., Nifelt Hagerstrom M., Blank U., Karlsson G. & Karlsson S. Human hematopoietic stem/progenitor cells overexpressing Smad4 exhibit impaired reconstitution potential *in vivo*. Blood 120, 4343–4351 (2012). 23018642 10.1182/blood-2012-02-408658

[b30] Grzywacz B. *et al.* Coordinated acquisition of inhibitory and activating receptors and functional properties by developing human natural killer cells. Blood 108, 3824–38338 (2006). 16902150 10.1182/blood-2006-04-020198 PMC1895469

[b31] Luevano M. *et al.* Frozen cord blood hematopoietic stem cells differentiate into higher numbers of functional natural killer cells *in vitro* than mobilized hematopoietic stem cells or freshly isolated cord blood hematopoietic stem cells. PLoS One 9, e87086 (2014). 24489840 10.1371/journal.pone.0087086 PMC3906137

[b32] Freud A. G. *et al.* Evidence for discrete stages of human natural killer cell differentiation *in vivo*. The Journal of experimental medicine 203, 1033–1043 (2006). 16606675 10.1084/jem.20052507 PMC2118285

[b33] Trzonkowski P., Szmit E., Mysliwska J., Dobyszuk A. & Mysliwski A. CD4+CD25+ T regulatory cells inhibit cytotoxic activity of T CD8+ and NK lymphocytes in the direct cell-to-cell interaction. Clin Immunol 112, 258–267 (2004). 15308119 10.1016/j.clim.2004.04.003

[b34] Carson W. E. *et al.* The functional characterization of interleukin-10 receptor expression on human natural killer cells. Blood 85, 3577–3585 (1995). 7540068

[b35] Huntington N. D. *et al.* IL-15 trans-presentation promotes human NK cell development and differentiation *in vivo*. J Exp Med 206, 25–34 (2009). 19103877 10.1084/jem.20082013 PMC2626663

[b36] Guichelaar T. *et al.* Human regulatory T cells do not suppress the antitumor immunity in the bone marrow: a role for bone marrow stromal cells in neutralizing regulatory T cells. Clin Cancer Res 19, 1467–1475 (2013). 23382115 10.1158/1078-0432.CCR-12-2177

[b37] Fujisaki J. *et al.* *In vivo* imaging of Treg cells providing immune privilege to the haematopoietic stem-cell niche. Nature 474, 216–219 (2011). 21654805 10.1038/nature10160 PMC3725645

[b38] Müller A. M. *et al.* Donor hematopoiesis in mice following total lymphoid irradiation requires host T-regulatory cells for durable engraftment. Blood 123, 2882–2892 (2014). 24591203 10.1182/blood-2013-10-530212 PMC4007614

[b39] Castriconi R. *et al.* Transforming growth factor beta 1 inhibits expression of NKp30 and NKG2D receptors: consequences for the NK-mediated killing of dendritic cells. Proceedings of the National Academy of Sciences of the United States of America 100, 4120–4125 (2003). 12646700 10.1073/pnas.0730640100 PMC153058

[b40] Kale V. P. Differential activation of MAPK signaling pathways by TGF-beta1 forms the molecular mechanism behind its dose-dependent bidirectional effects on hematopoiesis. Stem Cells Dev 13, 27–38 (2004). 15068691 10.1089/154732804773099236

[b41] Kale V. P. & Vaidya A. A. Molecular mechanisms behind the dose-dependent differential activation of MAPK pathways induced by transforming growth factor-beta1 in hematopoietic cells. Stem Cells Dev 13, 536–547 (2004). 15588511 10.1089/scd.2004.13.536

[b42] Gascoyne D. M. *et al.* The basic leucine zipper transcription factor E4BP4 is essential for natural killer cell development. Nature immunology 10, 1118–1124 (2009). 19749763 10.1038/ni.1787

[b43] Colucci F. Differential requirement for the transcription factor PU.1 in the generation of natural killer cells versus B and T cells. Blood 97, 2625–2632 (2001). 11313251 10.1182/blood.v97.9.2625

[b44] Boos M. D., Yokota Y., Eberl G. & Kee B. L. Mature natural killer cell and lymphoid tissue-inducing cell development requires Id2-mediated suppression of E protein activity. The Journal of experimental medicine 204, 1119–1130 (2007). 17452521 10.1084/jem.20061959 PMC2118569

[b45] Yun S. *et al.* TOX regulates the differentiation of human natural killer cells from hematopoietic stem cells *in vitro*. Immunology letters 136, 29–36 (2011). 21126536 10.1016/j.imlet.2010.11.008

[b46] Nguyen V. H. *et al.* The impact of regulatory T cells on T-cell immunity following hematopoietic cell transplantation. Blood 111, 945–953 (2008). 17916743 10.1182/blood-2007-07-103895 PMC2200838

[b47] Brunstein C. G. *et al.* Adoptive transfer of umbilical cord blood-derived regulatory T cells and early viral reactivation. Biology of blood and marrow transplantation: journal of the American Society for Blood and Marrow Transplantation 19, 1271–1273 (2013). 10.1016/j.bbmt.2013.06.004 PMC3870093 23806771

[b48] Jaatinen T. & Laine J. Isolation of hematopoietic stem cells from human cord blood. Curr Protoc Stem Cell Biol Chapter 2, Unit 2A 2, doi: 10.1002/9780470151808.sc02a02s1 (2007). 18785174

[b49] Figueroa-Tentori D., Querol S., Dodi I. A., Madrigal A. & Duggleby R. High purity and yield of natural Tregs from cord blood using a single step selection method. J Immunol Methods 339 (2008). 10.1016/j.jim.2008.09.019 18950634

[b50] Bohdan B. HeatmapGenerator: High performance RNAseq and microarray visualization software suite to examine differential gene expression levels using an R and C++ hybrid computational pipeline. Source Code for Biology and Medicine 9, doi: 10.1186/s13029–014–0030–2 (2014). PMC4279803 25550709

