# Supplementary material for: Regulatory T cells inhibit CD34+ cell differentiation into NK cells by blocking their proliferation
Source: Sci Rep. 2016 Feb 26;6:22097. doi: 10.1038/srep22097 (PMC4768165; doi:10.1038/srep22097)
Supplement: Supplementary Information [file srep22097-s1.pdf]

Supplemental information

Regulatory T cells inhibit CD34+ cell differentiation into NK cells by blocking their proliferation

Isabela Pedroza-Pacheco[1], Divya Shah[1], Anna Domogala[1], Martha Luevano[1], Michael Blundell[2], Nicola Jackson[1], Adrian Thrasher[2], Alejandro Madrigal[1] and Aurore Saudemont[1,*]

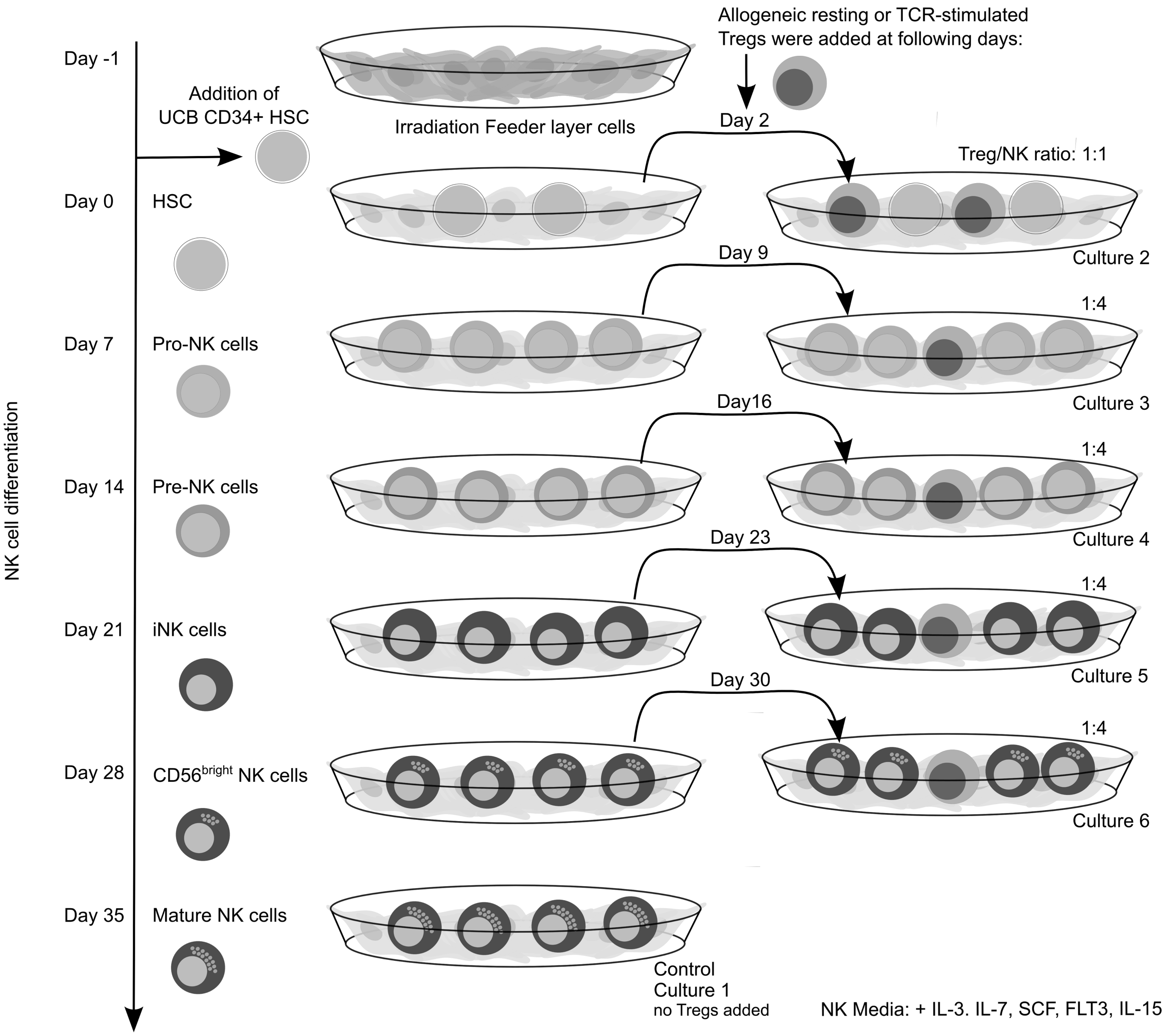

Figure S1: Experimental design used to assess the effect of Tregs on NK cell differentiation *in vitro.* HSC ± resting or activated Tregs were cultured in the presence of irradiated EL08.1D2 feeder layer cells and cytokines for 35 days. Tregs were added to HSC cultures at a 1:4 ratio (Tregs:NK cells), except when added at day 2, where a ratio of 1:1 was used. Activated Tregs were activated with plate bound anti-CD3/soluble anti-CD28 and 1000 IU/mL IL-2 for 24 h and washed before addition to HSC cultures.

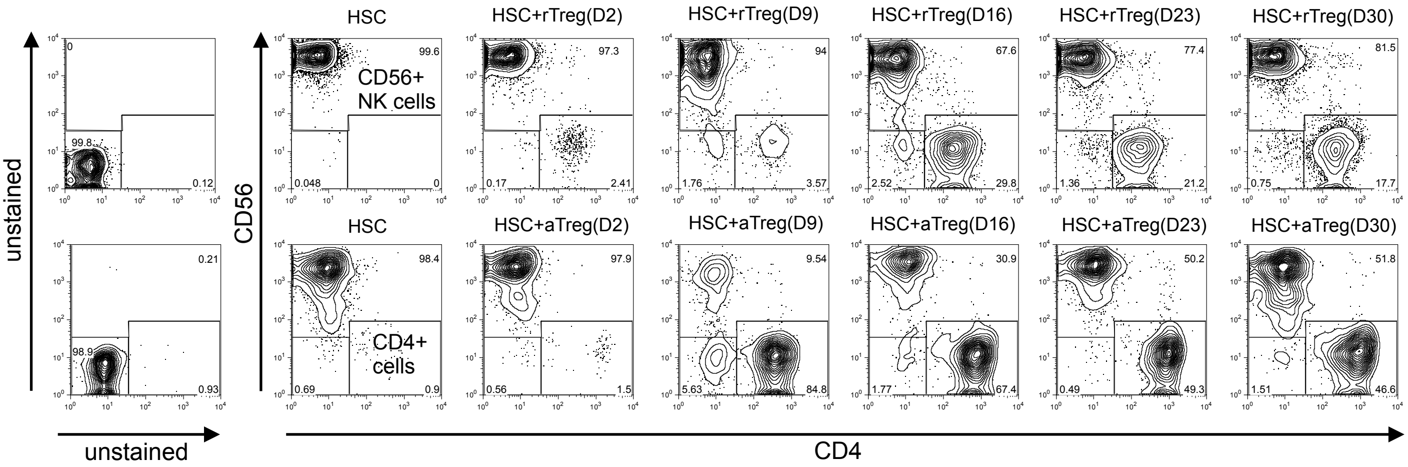

Figure S2: Percentages of NK cells and CD4+ cells in HSC cultures. HSC were cultured ± resting or activated Tregs added at days 2, 9, 16, 23 and 30 of differentiation at a 1:4 ratio (Tregs:HSC). Representative flow cytometric analysis of NK cells ($CD56^{+}$) and Tregs ($CD4^{+}$) from all cultures at day 35 of HSC cultures.

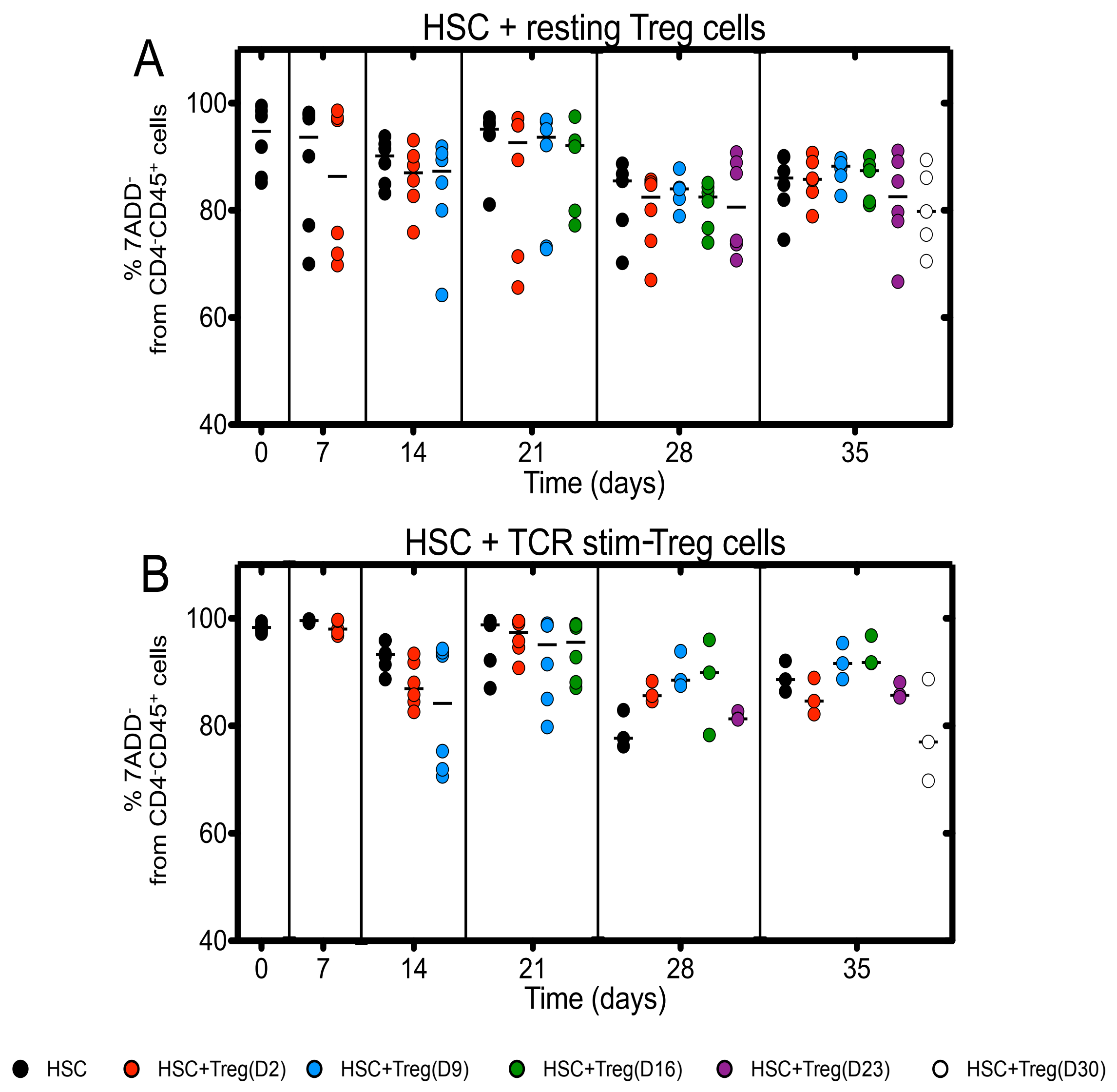

Figure S3: Viability of CD45$^+$ cells in HSC cultures in the presence or absence of resting or TCR-stimulated CB Treg cells. Viability of CD45$^+$ was assessed by flow cytometry using CD4, CD45 and 7-AAD. HSC were cultured in the presence or absence of allogeneic resting CB Treg cells (A) or TCR-stimulated CB Treg cells (B). Treg cells were added at key time points of differentiation. The lines represent medians. n=6-8.

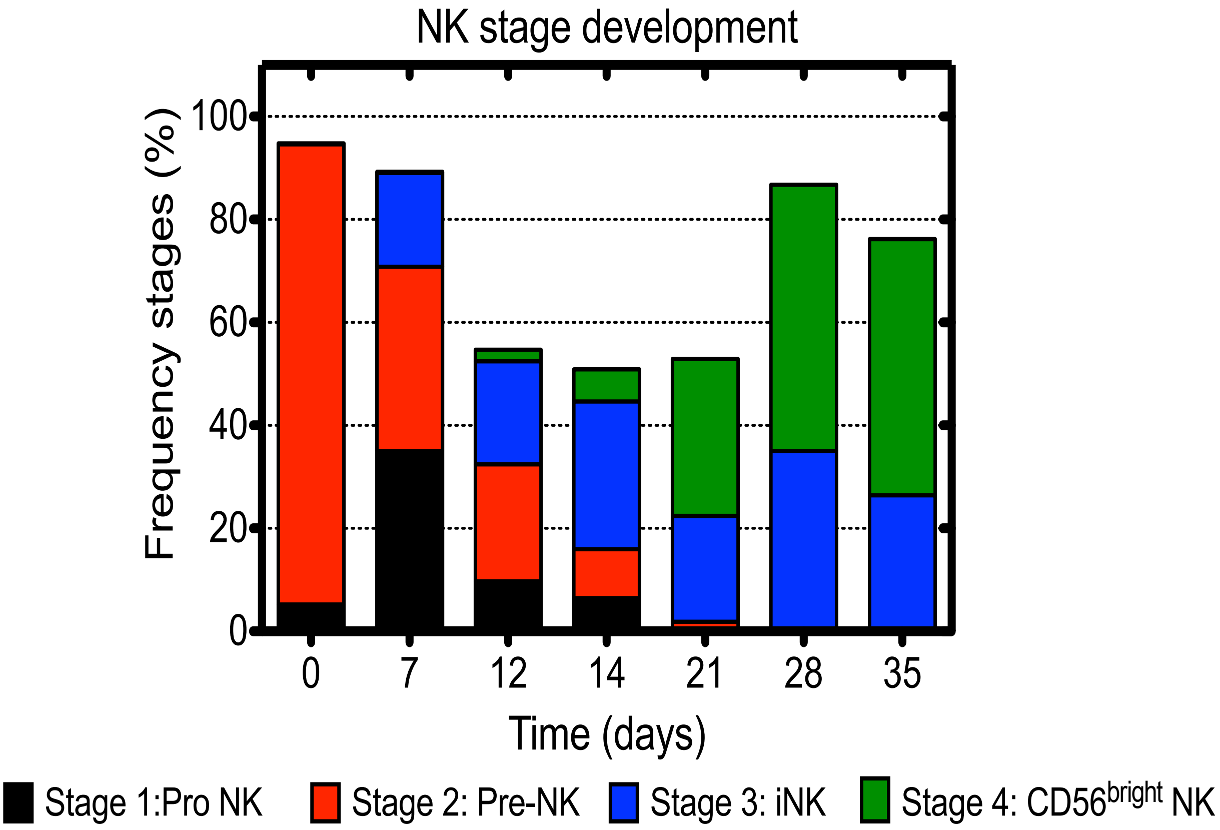

Figure S4: Frequency of cells in each NK cell differentiation stage in HSC cultures. HSC were cultured for 35 days and frequencies of Stage 1-4 NK cells were assessed based on CD4, CD34, CD117 and CD94 expression at days 0, 7, 14, 21, 28 and 35. The values represent medians. n=11

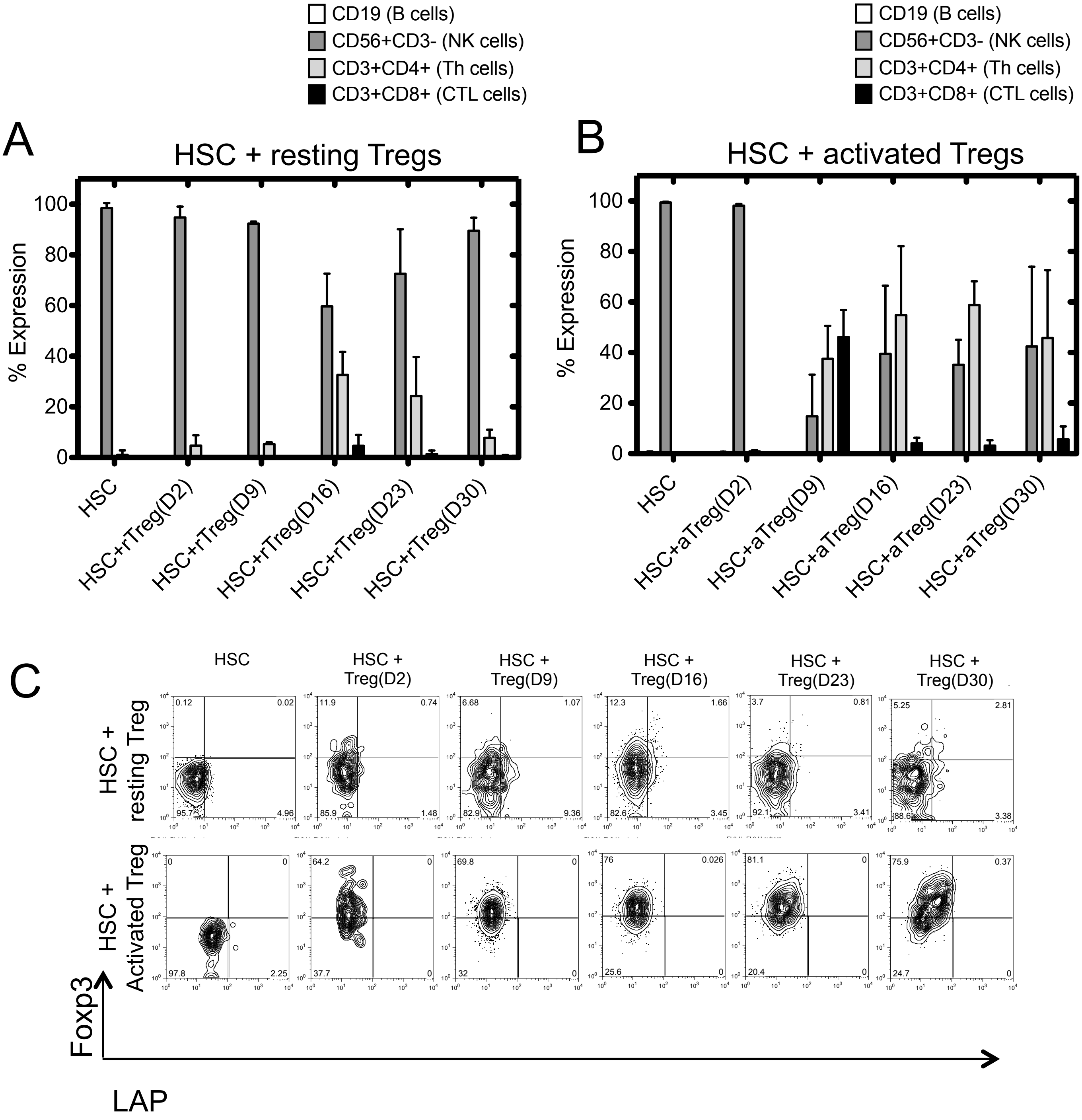

Figure S5: Phenotypic analysis of lymphocyte populations after HSC cultures. Flow cytometry analysis of CD4, CD8, CD19 and CD56 in HSC cultures with resting or activated Tregs (**A-B**). Expression of Foxp3 and LAP on CD4 T cell populations. HSC were used as negative control of expression (**C**).

Table S1. Primer sequences used for real time PCR. Conc. indicates concentration.

**Primer**	**Sequence**	**Conc (nM)**
**BCL11B**	F: 5'-CTCTCACCCACGAAAGGCAT-3'<br>R: 5'-GCACGCAGAGGTGAAGTGAT-3'	300
**E4BP4**	F: 5'-CCAAGGGCCCCATCCATTC-3'<br>R: 5'-GATGCCAGTGCTCCGATTTG-3'	300
**FOXP3**	F: 5'-CACCTGGCTGGGAAAATGG-3'<br>R: 5'-GGAGCCCTTGTCGGATGAT-3'	900
**GATA-3**	F: 5'-AGCACAGAAGGCAGGGAGTGT-3'<br>R: 5'-TTCGCTTGGGCTTAATGAGGGGC-3'	300
**HELIOS**	F: 5'-ACACCTCAGGACCCATTCTG-3′<br>R: 5'-TCCATGCTGACATTCTGGAG-3′	600
**ID2**	F: 5'-CGGATATCAGCATCCTGTCC-3′<br>R: 5'-TCATGAACACCGCTTATTCAG-3′	300
**PU.1**	F: 5'-TGTTACAGGCGTGCAAAATGGAAGG-3'<br>R: 5'-CTCGTGCGTTTGGCGTTGGTATAGA-3'	300
**RORC**	F: 5'-AGTCGGAAGGCAAGATCAGA-3'<br>R: 5'-CAAGAGAGGTTCTGGGCAAG-3'	300
**T-BET**	F: 5'-GGATGCGCCAGGAAGTTTCA-3'<br>R: 5'-CTCTGGCTCTCCGTCGTTCA-3'	300
**TOX**	F: 5'-TATGTGCCAGCCAGCCAGTCCTA-3'<br>R: 5'-TGGTCTGGGAGGGAAGGAGGAGTAA-3'	300

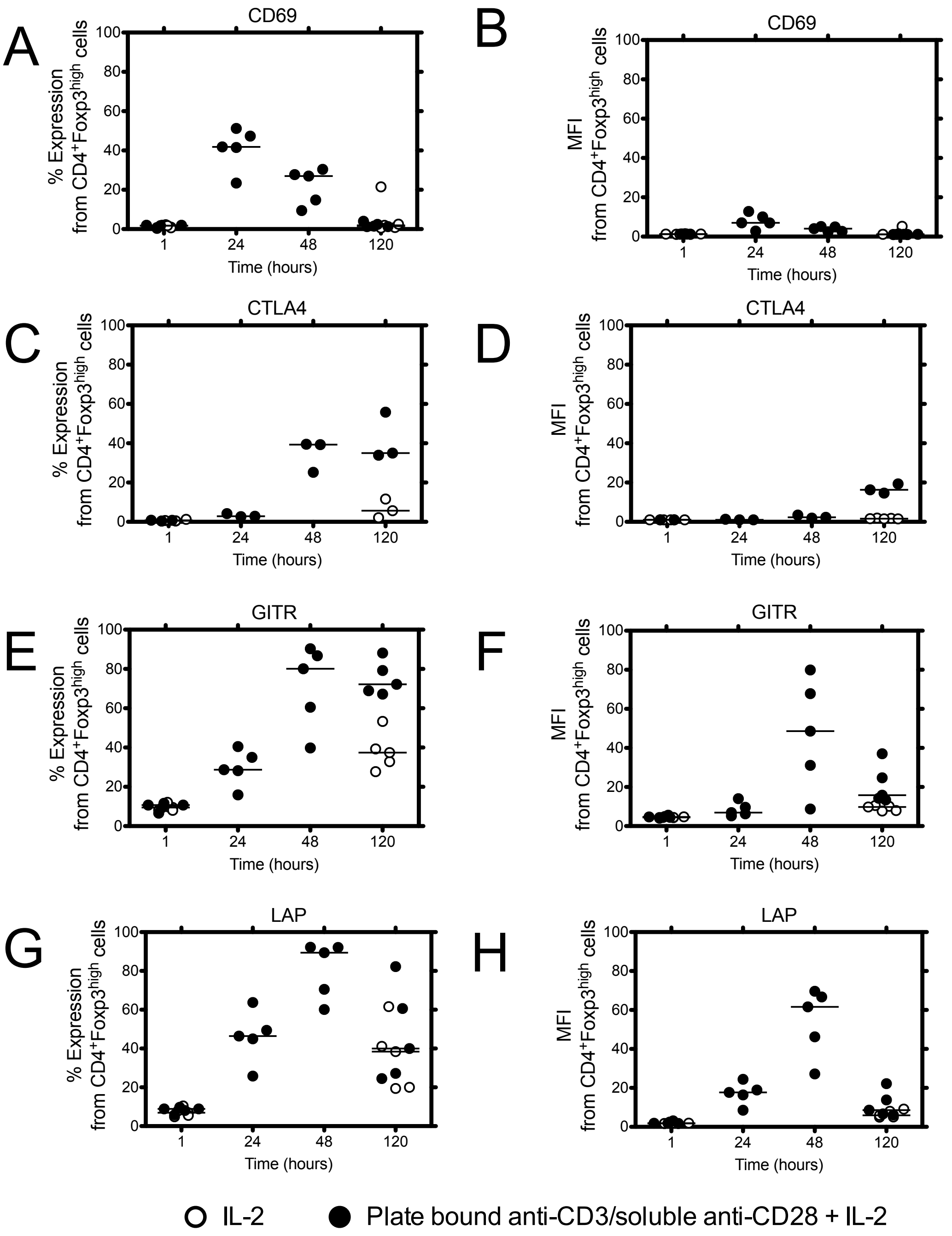

Figure S6: Phenotypic analysis of Tregs after activation. Flow cytometric analysis of Tregs during stimulation with plate bound anti-CD3/soluble anti-CD28 and 1000 IU/mL IL-2. Frequency of expression and MFI of CD69 (**A-B**), CTLA4 (**C-D**), GITR (**E-F**) and LAP (**G-H**) were assessed on gated $CD4^{+}Foxp3^{high}$ Tregs. n = 3-5.
